# Broadly Targeted Metabolomics Analysis of Differential Metabolites Between *Bupleurum chinense* DC. and *Bupleurum scorzonerifolium* Willd.

**DOI:** 10.3390/metabo15020119

**Published:** 2025-02-11

**Authors:** Min Li, Quanfang Zhang, Tongshan Zhu, Guoxia Liu, Wenxiao Chen, Yanli Chen, Xun Bu, Zhifeng Zhang, Yongqing Zhang

**Affiliations:** 1 State Key Laboratory of Quality Research in Chinese Medicine, Faculty of Chinese Medicine, Macau University of Science and Technology, Macau 999078, China; 2109853dcw30004@student.must.edu.mo; 2 College of Pharmacy, Shandong University of Traditional Chinese Medicine, Jinan 250355, China; zhangquanfang@saas.ac.cn (Q.Z.);; 3 Key Laboratory of Traditional Chinese Medicine Classical Theory, Ministry of Education, Shandong University of Traditional Chinese Medicine, Jinan 250355, China; 4 Institute of Crop Germplasm Resources, Shandong Academy of Agricultural Sciences, Jinan 250131, China

**Keywords:** *Bupleurum chinense* DC., *Bupleurum scorzonerifolium* Willd., differentially accumulated metabolites, Chinese medicine, UPLC/ESI-Q TRAP-MS/MS

## Abstract

**Background/Objectives:** Bupleuri Radix is a plant in the Apiaceae family *Bupleurum Chinense* DC. or *Bupleurum scorzonerifolium* Willd. root. The dissimilarities in the metabolite profiles of plants directly correlate with the disparities in their clinical efficacy. **Methods**: Therefore, the wild *Bupleurum Chinense* DC. (YBC) and wild *Bupleurum scorzonerifolium* Willd. (YNC) were used as research materials. They were analyzed using the UPLC-MS/MS and the similarities and differences were uncovered based on differential metabolites. **Results:** Our results proved that the differences in clinical efficacy between YBC and YNC may be attributed to their distinct metabolite profiles, as follows: (1) a total of 12 classes of 2059 metabolites were identified in the roots, with phenolic acids, terpenoids, and flavonoids being the most abundant metabolic products, with 2026 shared components between the two, 2045 in YBC, and 2040 in YNC; (2) a total of 718 differential metabolites were identified, accounting for 35.44% of the shared metabolites. Among them, YBC had 452 metabolites with higher content relative to YNC, representing 62.95%, and 266 components with lower content, representing 37.05%; (3) the KEEG enrichment analysis results show that the differential metabolic pathways are flavone and flavonol biosynthesis, linoleic acid metabolism, arachidonic acid metabolism, sesquiterpenoid and triterpenoid biosynthesis, and linolenic acid metabolism. **Conclusions:** These new findings will serve as a foundation for further study of the BR biosynthetic pathway and offer insights into the practical use of traditional Chinese medicine in clinical settings.

## 1. Introduction

Bupleuri Radix (BR, or Chaihu in Chinese) is a plant in the Apiaceae family *Bupleurum Chinense* DC. (BC) or *Bupleurum scorzonerifolium* Willd. (NC) dried root. According to their different characters, they were called “Northern Bupleurum” and “Southern Bupleurum” and had a medicinal history of more than 2000 years [1]. BR contains saponins, flavonoids, volatile oils, polysaccharides, lignans and other components [2]. It has the effect of clearing heat deficiency [3], soothing the liver and relieving depression [4], and lifting Yang Qi [5], and was often used for cold fever, cold and heat exchanges, chest and hypochondriac pain, uterine prolapse, menstrual irregularity, insomnia, and dreaminess [6,7]. On the other hand, it has anti-tumor, anti-depression [8], anti-inflammatory, anti-viral, and other effects [9]. It was a commonly used clinical medication for relieving exterior symptoms and has a large market demand.

In the clinical application, BC is mainly used to treat typhoid fever and NC is mainly used to clear liver fever [10]. Over the past decade, in most areas of our country, there was no distinction and there was confusion about its use. Some parts of China, such as Beijing, required that when using two kinds of BR, the doctors should prescribe them separately as needed. It is traditionally believed that NC has better quality than BC because of its medicinal material properties, color, odor, and habitat [11,12,13]. However, the quality of medicinal materials is uneven, high-quality BR resources were relatively scarce, and BC was the mainstream in the north of commodity circulation. Currently, the medicinal materials mainly come from cultivated bupleurum, mainly from Gansu, Shanxi, and Shaanxi provinces.

Studies have shown that there were differences in pharmacological effects between them. Through the study of the saikosaponin (SS) content in NC and BC, the results showed that the total SS in BC and SSa were more than those in NC, and some studies showed that the total SS in BC was about four times that in NC [14]. In addition, through the study of the volatile oil components in BR, it was found that the volatile oil content of BC was much lower than that of NC, and there were obvious differences in the chemical composition [15]. Not only were there many differences in the proportion of common components, but NC also contained some unique sesquiterpene components, such as piperene, A-cedrene, b-elemene, etc. Guo et al. [16] compared the antidepressant efficacy of different varieties of Xiao Yao powder composed of BR to explore its influence on the changes in endogenous metabolites in rats, and found that both had obvious antidepressant effects, but the antidepressant effect and onset time of Xiaoyao powder composed of NC was slightly better than that of BC, indicating that NC had a unique advantage in pharmacological activity.

Metabolomics technology can comprehensively analyze metabolites in organisms, provide strong technical support for the study of natural products, and provide a more comprehensive perspective for the study of natural products [17]. It’s a newly developed important subject of bioomics and also is an important component of systems biology [18,19,20]. In this study, we selected the wild *Bupleurum chinense* DC. (YBC) and *Bupleurum scorzonerifolium* Willd. (YNC) as research materials, and the metabolite types of them were detected by using widely targeted metabolomics methods and ultra-high-performance liquid chromatography tandem mass spectrometry (UPLC/ESI-Q TRAP-MS/MS) technology, and their differences were compared and analyzed. From the perspective of different metabolites, the similarities and differences between them were revealed, which provided references for the development and utilization of BR.

## 2. Materials and Methods

### 2.1. Plant Materials

**Figure 1:**
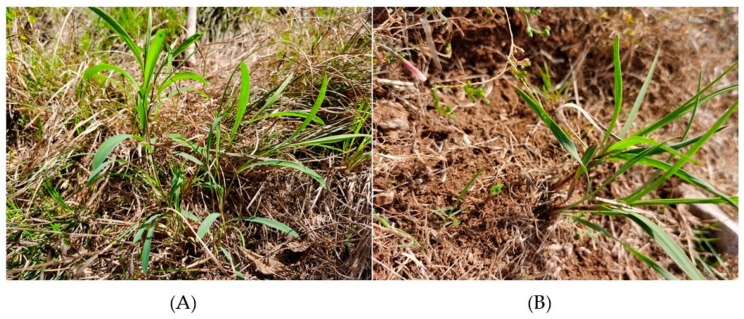
The living environment of YNC and YBC at the time of sample collection ((**A**):YBC; (**B**):YNC).

In this study, all the selected samples were two-year-old wild resources from the suburban mountains areas of Yiyuan County [Zibo, Shandong, China (Lat. 36°09′ N, 118°34′ E/36°20′ N, 118°28′ E)] in April 2023 (Figure 1). The original plants of the samples were identified as umbelliferae plants *Bupleurum chinense* DC. (YBC) and *Bupleurum scorzonerifolium* Willd. (YNC) [21,22]. The work was carried out by Professor Yongqing Zhang from Shandong University of Traditional Chinese Medicine, and the samples were stored in the ultra-low temperature refrigerator of Shandong Academy of Agricultural Sciences. Materials were taken for 3 repetitions with each 5 mL freezer tube, then quickly placed in liquid nitrogen. In addition, all samples were transferred to an ultra-low-temperature refrigerator at −80 °C for storage.

### 2.2. Sample Preparation and Extraction

Sample preparation and extraction were performed based on the methods which were provided by the Metware Biotechnology Co., Ltd. (Wuhan, China) exactly as previously described [23]. In total, 6 samples are dehydrated using a vacuum freeze-drying apparatus ((Scientz-100F, Ningbo, China). The freeze-dried samples were crushed using a mixer mill ((MM400, Retsch, Haan, Germany) with a zirconia bead for 1.5 min at 30 Hz. Then, 50 mg lyophilized powder of each sample was weighed and was dissolved in 1.2 mL 70% methanol solution, vortexed for 30 s at 30 min intervals, totaling 6 vortexing sessions, and the samples were placed in a refrigerator at 4 °C overnight. After extraction, the mixtures were centrifuged at 16,260 g for 10 min, and then the supernatant was collected and filtered through a micropore filter membrane (SCAA-104, 0.22 μm pore size; ANPEL, Shanghai, China). The obtained filtrates were stored in a vial for ultra-high-performance liquid chromatography tandem mass spectrometry (UPLC-MS/MS) analysis.

### 2.3. UPLC Conditions

Sample extract solutions were analyzed in the UPLC-ESI-MS/MS system (HPLC, Shim-pack UFLC SHIMADZU CBM30A system; MS, Applied Biosystems 6500 QTRAP). The analysis conditions were as follows: UPLC, column, Agilent SB-C18 (1.8 µm, 2.1 mm × 100 mm); mobile phase, solvent A (pure water with 0.1% formic acid) and solvent B (acetonitrile with 0.1% formic acid); gradient elution, 95–5% A at 0–9 min, 5% A at 9–10 min, 5–95% A at 10–11.1 min, and 95% A at 11.1–14 min; flow rate, 0.35 mL per minute; temperature, 40 °C; and injection volume, 4 μL. The effluent was alternatively connected to an ESI-triple quadrupole-linear ion trap (QTRAP)-MS.

### 2.4. ESI-Q TRAP-MS/MS

ESI source operational parameters, included the source temperature of 500 °C; ion spray voltage (IS) 5500 V (positive ion mode)/−4500 V (negative ion mode); ion source gas I (GSI), gas II (GSII), and curtain gas (CUR) were set at 50, 60, and 25 psi, respectively; the collision-activated dissociation (CAD) was high. QQQ scans were acquired as MRM experiments with collision gas (nitrogen) set to medium. DP (declustering potential) and CE (collision energy) for individual MRM transitions were conducted with further DP and CE optimization. A specific set of MRM transitions was monitored for each period according to the metabolites eluted within the respective period.

### 2.5. Qualitative and Quantitative Determination of Metabolites

For the identification and characterization of metabolites, the primary and secondary MS data were used to annotate metabolites based on the self-built metware database (MWDB) (Wuhan Metware Biotechnology Co., Ltd., Wuhan, China) [24]. To ensure the accuracy of the experimental results, the isotopic signal included the repeated signals of K^+^, Na^+^, and NH4^+^ ions. Additionally, during the analysis, the recurring signals from fragments of ions with higher molecular weight were eliminated. Metabolite quantification was performed using multiple reaction monitoring (MRM) by triple quadrupole mass spectrometry. In the MRM model, the four-pole first screens the precursor ions (parent ions) of the target substance and excludes the ions corresponding to other molecular weight substances to preliminarily eliminate the interference. After ionization induced by the collision chamber, the precursor ion breaks to form a lot of fragment ions, and then a characteristic fragment ion is selected through the triple-quadrupole filtering to remove the interference from non-target ions, thereby enhancing the accuracy of quantification and improving repeatability [25].

### 2.6. Quality Control Sample Analysis

The quality control (QC) samples were created by combining equal amounts of ex-tracts from YNC and YBC, with three replicates. The same method was used for processing and testing as for the analysis samples. During the instrument testing process, one quality control sample was introduced into every ten detection analysis samples to oversee the repeatability of the entire analysis process. By comparing the total ion current (TIC) chromatograms of different quality control samples obtained through mass spectrometry analysis, the repeatability of metabolite extraction and detection can be determined, this can also be considered as technical repeatability. Therefore, the high stability of the instrument serves as a crucial safeguard for the repeatability and reliability of the data.

### 2.7. Statistical Analysis

After the metabolic mass spectrometry data of different samples were obtained, the peak area integral of all chromatographic peaks was performed, and the mass spectrum peak of the same metabolite in different samples was integrated and corrected. The mass spectrometry data were processed using Analyst 1.6.3 software, and principal component analysis (PCA) and cluster analysis of the two sample groups were performed using multivariate statistical analysis methods. The stability and reliability of the model were assessed through partial least squares discriminant analysis (PLS-DA) and orthogonal partial least squares discriminant analysis (OPLS-DA). Different metabolites were screened by variable importance in project (VIP) values, unidimensional statistical *p*-values, and differential multiples. The heatmap program in R (v3.3.2) was used for cluster analysis of the selected differential metabolic components, and a heatmap was drawn [26]. The differential metabolites of the samples were selected by stratigraphy cluster, and the results were uploaded to the Kyoto Encyclopedia of Genes and Genomes (KEGG) database website for pertinent pathway analysis.

## 3. Results

### 3.1. Quality Control Sample Analysis

**Figure 2:**
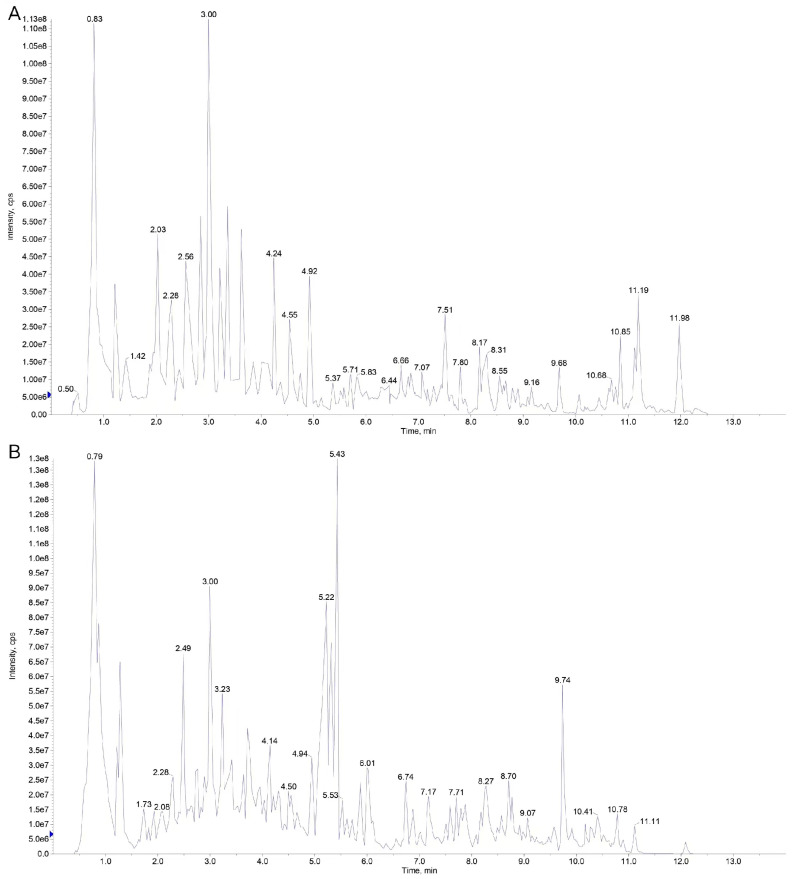
Total ion current plot for quality control. **Note:** (**A**) negative ion mode; (**B**) positive ion mode.

According to the (+) and (−) total ion flow diagram of the quality control sample (Figure 2), under this detection condition, the peak shape distribution of the total ion current (TIC) chromatogram of the quality control sample under positive and negative ion modes is relatively uniform and in good shape, indicating that the detection process is relatively stable and the detection results are authentic and reliable.

### 3.2. Metabolite Detection

In this study, a total of 2059 metabolites of 12 classes were identified (Table 1), which included 324 phenolic acids, 270 terpenoids, 254 flavonoids, 192 amino acids and derivatives, 191 lipids, 176 alkaloids, 173 lignans and coumarins, 120 organic acids, 72 nucleotides and derivatives, 16 quinones, 3 tannins, and 268 other metabolites.

**Figure 3:**
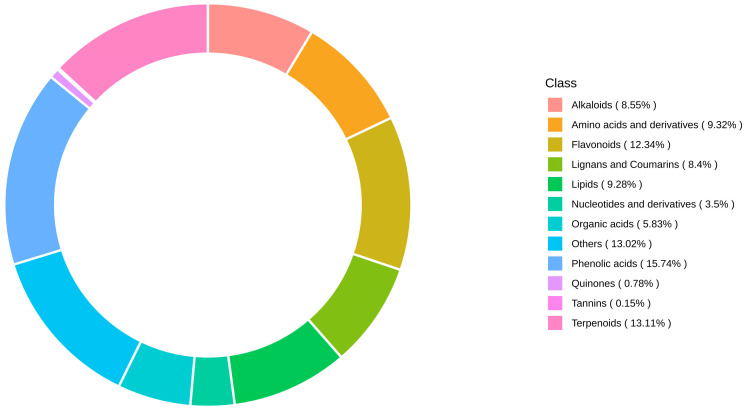
Circular diagram of metabolite class composition.

The number of metabolites was far more than the previous identified metabolite numbers. A circular diagram of metabolite classes is shown in Figure 3; these suggest that the UHPLC-QQQ-MS based targeted metabolomics method was a widely effective method for the comprehensive identification of metabolites in plants. In general, among these metabolites, 2026 existed in both, mainly phenolic acids, terpenes, flavonoids, amino acids, and their derivatives. These metabolites were listed in Table 1.

### 3.3. Analysis of Metabolomics Difference Between YBC and YNC by Multivariate Analysis

**Figure 4:**
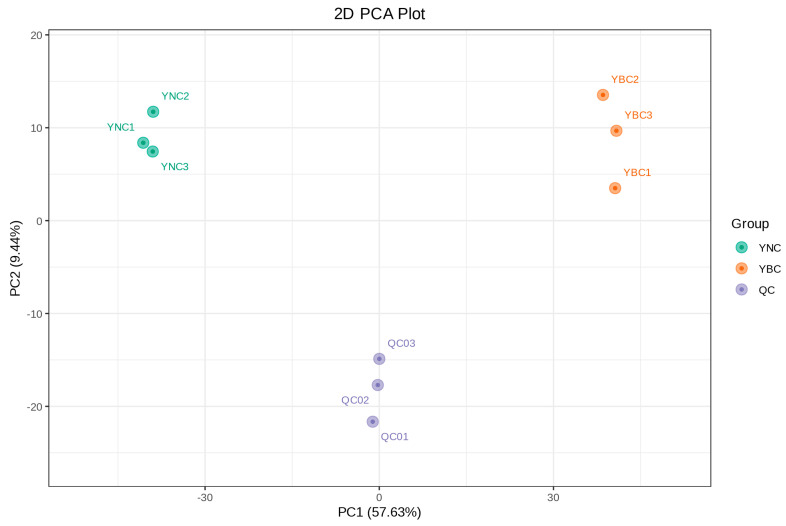
PCA score plot of the metabolites in the YBC and YNC.

Principal component analysis (PCA) can reflect the abundance of metabolites in samples. The closer the location, the greater the similarity, and the farther away, the smaller the similarity [27]. Through PCA analysis of the samples, the variation degree between the samples of YBC and YNC and between the samples in the group was determined. Five principal components were obtained, in which the contribution rate of PC1 was 57.63% and that of PC2 was 9.44%. The two groups of samples showed an obvious separation trend (Figure 4), and the separation of YBC was slightly greater than that of YNC. This may be due to their different living environments, which lead to differences in metabolites. The medicinal materials grown in the wild have a different quality and composition of medicinal materials, and the clinical treatment effect is better [28]. PCA clearly grouped these samples into distinct clusters, which indicates the significant differences in metabolites between the YBC and YNC.

**Figure 5:**
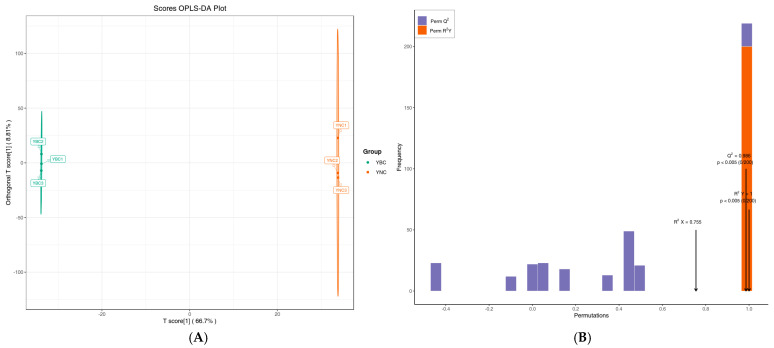
The score chart (**A**) and verification chart (**B**) of OPLS-DA.

PCA was capable of efficiently extracting primary information, yet it lacked sensitivity to variables that have low correlation. In contrast, partial least squares discriminant analysis (PLS-DA) addresses this issue and optimizes the separation between different groups; therefore, it is beneficial in searching for differential metabolites [29]. Orthogonal partial least squares discriminant analysis (OPLS-DA) integrates orthogonal signal correction (OSC) with PLS-DA, enabling the identification of differential variables by eliminating uncorrelated variations. Based on the OPLS-DA model (Figure 5A), 2059 metabolomics were analyzed: YBC was distributed on the left side of the confidence interval, and YNC was distributed on the right. The difference between the two samples was very obvious. The contribution rate of PC1 obtained by OPLS-DA was 66.7%, that of PC2 was 8.81%, R^2^X = 0.755, R^2^Y = 1, Q^2^ = 0.986, in which Q^2^ > 0.9, was an excellent model, which was better than the PCA model. For OPLS-DA, the arrangement verification (*n* = 200) was carried out. In the model verification (Figure 5B), the horizontal coordinate represented the values of R^2^Y and Q^2^ of the model, and the vertical coordinate was the frequency of the model classification effect in 200 random arrangement and combination experiments. The findings indicate that the model was significant, and its differential metabolites could be assessed and selected based on the VIP value.

Based on the results of the OPLS-DA model, the differential metabolites in YBC and YNC were screened. The screening criteria were as follows: (1) metabolites with VIP values > 1 were selected. The VIP value reflects the impact of the differences among the corresponding metabolites on the classification and discrimination of each group within the model. It is generally believed that metabolites with VIP value > 1 are significantly different; (2) a fold-change score ≥ 2 or ≤0.5 among the metabolites with a VIP value > 1 was used to identify differential metabolites. If the difference in metabolites between the control group and the experimental group is more than 2 times or less than 0.5 times, the difference is considered significant. A total of 11 types of metabolites with 718 significant differences were identified (Table 1); of these, differential metabolites accounted for 35.44% of the total metabolic components (2026 types), indicating that there were differences in metabolites between YBC and YNC. Flavonoids, phenolic acids, terpenoids, lignans, and coumarins were significantly different; the proportions were 19.22%, 18.52%, 16.30%, and 11.28%, respectively.

### 3.4. Different Metabolite Analyses

**Figure 6:**
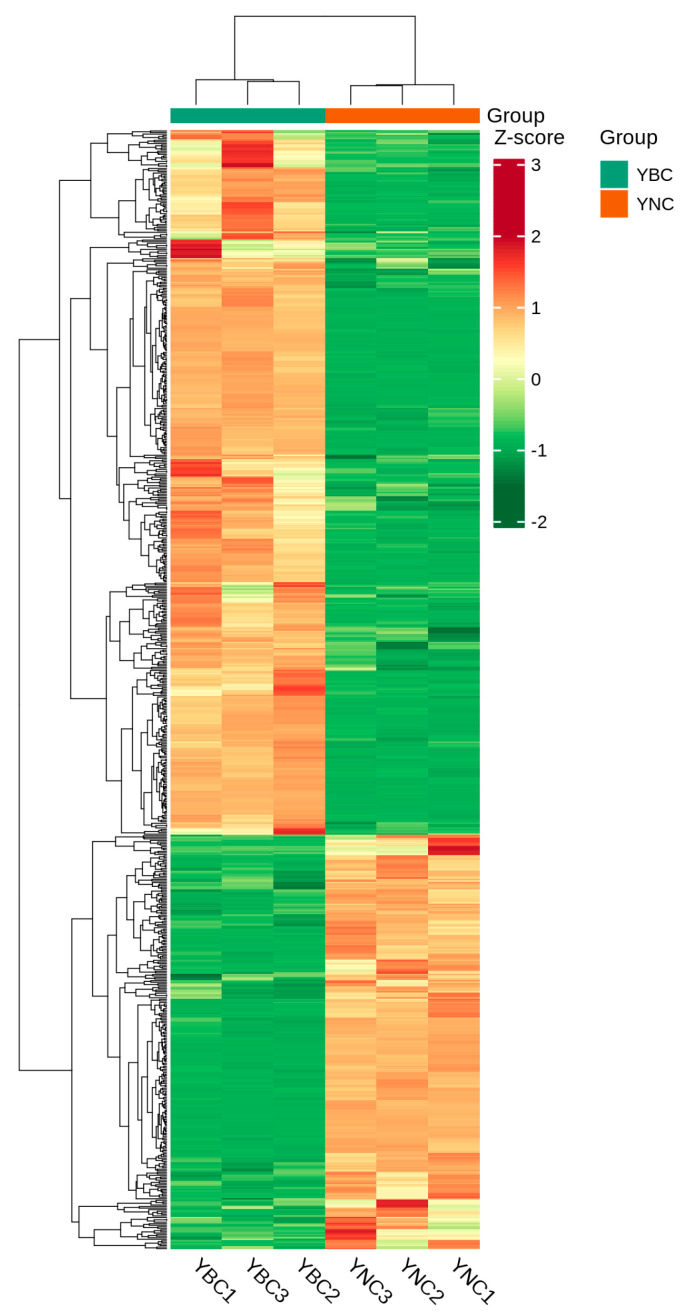
Heat map for differential metabolites.

To better visualize the patterns of metabolite changes, metabolites showing significant differences were normalized, and a clustering heatmap was generated. The results of all detected metabolites are shown in a heatmap (Figure 6). Which simply and intuitively reflects the changes in metabolites. Among the 718 differential metabolic components, 266 substances of YBC were down-regulated compared with YNC; that is, the relative content of YBC was decreased, and the decreased metabolic components accounted for 37.05% of the differential metabolic components; 452 substances were up-regulated; that is, the relative content increased, and the increased metabolic components accounted for 62.95% of the differential metabolic components. The difference in metabolite type and content between YBC and YNC resulted in the difference in pharmacodynamic composition.

The fold changes in the quantitative data of metabolic constituents in YBC and YNC were compared and the difference multiples were processed (log_2_FC). The top 20 differentially expressed metabolic components with changes are shown in Table 2. Compared with YNC, the relative contents of five flavonoids (Kaempferol-3-O-rhamnoside(Afzelin) (Kaempferin), 8-Methoxykaempferol-7-O-rhamnoside, Kaempferol-7-O-rhamnoside, Hispidulin-7-O-(6″-O-p-Coumaroyl)Glucoside,Quertin-3,7-Di-O-rhamnoside),3phenolicacids(3,5-dihydroxy-4-{[(2s,3r,4s,5s,6r)-3,4,5-trihydroxy-6-[(sulfooxy)methyl]oxan-2-yl]oxy}benzoic acid,3,5-dihydroxy-4-[(2S,3R,4S,5S,6R)-3,4,5-trihydroxy-6-(sulfooxymethyl)oxan-2-yl]oxybenzoic acid, 5-O-Feruloylquinic acid), three lignans and coumarins (5,7-Dimethoxycoumarin (Limettin) (Citropten), 6,7Dimethoxy-4-methylcoumarin, 3-Methyl-6-methoxy-8-hydroxy-3,4-dihydroisocoumarin), and three other substances (Eugenin, Leptorumol, and 30-O-Aangeloylhamaudol) in YBC root were significantly increased. The relative contents of two alkaloids (Hydroxystemofoline and Cinnamoyltyramine), two terpenoids (Ajugamacrin C* and Ajugamacrin D*), one phenolic acid (Vanillin acetate), and one quinone (6-Methylaloe emodin) were significantly reduced.

### 3.5. Kyoto Encyclopedia of Genes and Genomes Annotation and Enrichment Analysis

**Figure 7:**
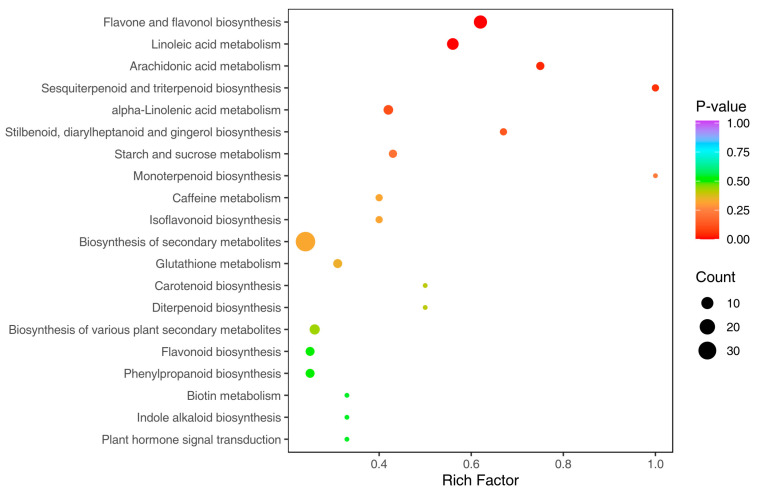
KEGG enrichment map for differential metabolites **Note:** The color of the points is a *p*-value; the redder the color, the more significant the enrichment. The size of the points represents the number of enriched differentiated metabolites.

**Figure 8:**
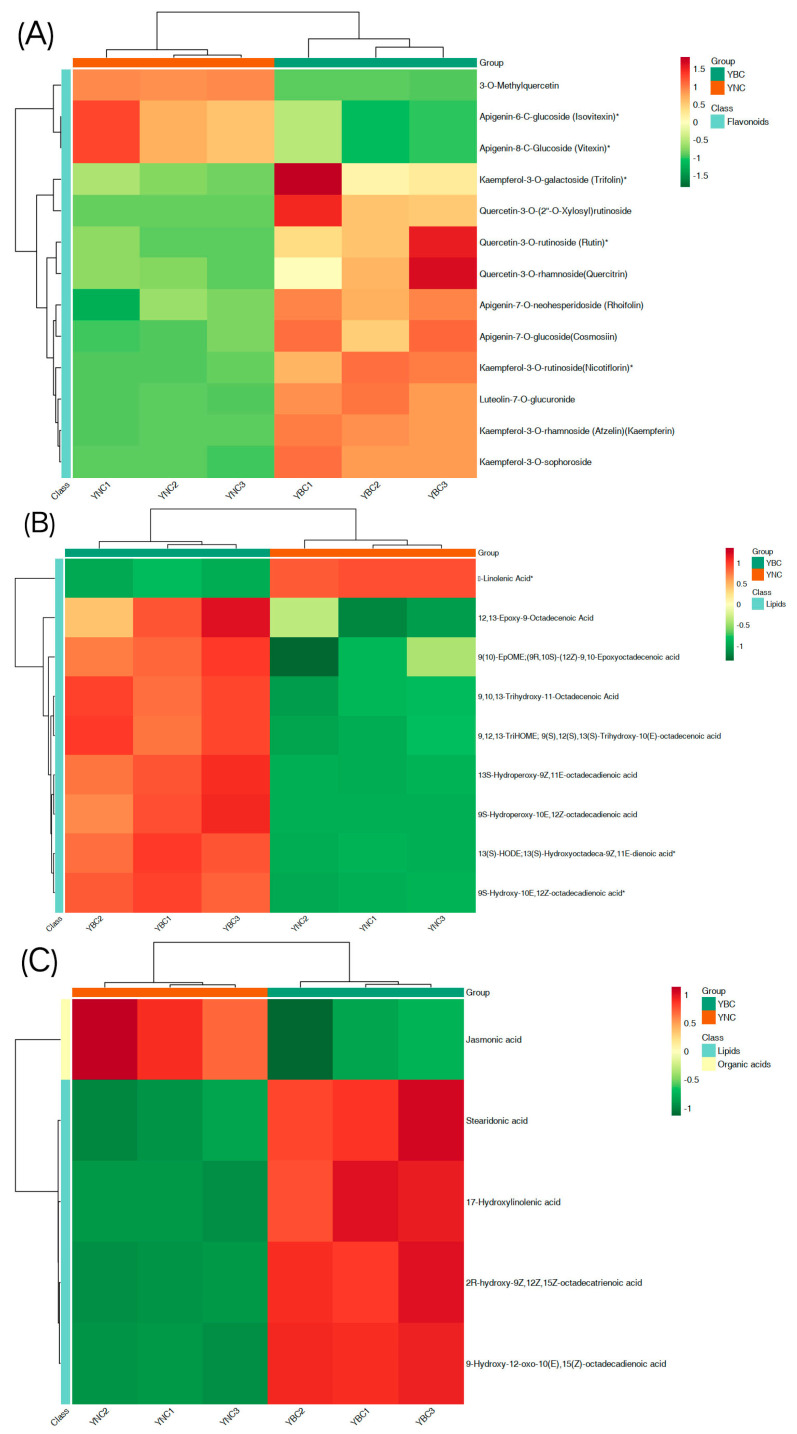
Heat map depicting the differential metabolite content within related metabolic pathways. **Note:** (**A**) Flavone and flavanol biosynthesis; (**B**) linoleic acid metabolism; (**C**) alpha-linolenic acid metabolism. If there are less than 5 differential metabolites in the pathway, the pathway is not displayed.

The KEGG database was employed to carry out pathway enrichment analysis of differential metabolites; significant metabolic pathways can be mined [30]. The results are shown in Figure 7, in which 20 enrichment pathways with the lowest *p* value are shown (Figure 7, Table 3), among which, 93 metabolites with significant differences have been noted. The metabolic pathways with significant differences are flavonoid and flavanol biosynthesis, linoleic acid metabolism, arachidonic acid metabolism synthesis, sesquiterpenoid and triterpenoid biosynthesis, and linolenic acid metabolic synthesis pathways. A total of 32 metabolites are involved in these five pathways, mainly costunolide are γ-Linolenic Acid,3-O-Methylquercetin, 8,9-Dihydroxy-5Z,11Z,14Z-eicosatrienoic acid, and Jasmonic acid (Figure 8).

## 4. Discussion

In recent years, BR has been a wide concern, and large-scale cultivation has been carried out in Gansu, Shanxi, Inner Mongolia and other regions. Nowadays, the research on BR mainly focuses on BC, but there was little focus on NC [31]. In this study, the metabolites of YBC and YNC were compared and analyzed using widely targeted metabolomics technology. In total, 2059 metabolites were recognized in the two BRs, among 324 phenolic acids, 270 terpenes, 254 flavonoids, 192 amino acids and their derivatives, and other compounds. Among them, 718 differential metabolites were screened out, and the differential metabolites (718) accounted for 35.44% of the total metabolites (2026), indicating that the metabolite profiles of the two groups were distinct. The major differences were in flavonoids, phenolic acids, terpenes, lignans, and coumarins, accounting for 19.22%, 18.52%, 16.30% and 11.28%, respectively, the differences in these metabolites may be related to the differences in the efficacy of the two drugs. Studies have shown that the differential metabolites in the aboveground parts were primarily associated with monoterpenoid biosynthesis pathways, whereas those in the roots were mainly linked to sesquiterpenoid and triterpenoid biosynthesis pathways [32]. And the superior anti-inflammatory properties of NC can be attributed to its distinct chemical composition compared to BC, as revealed by GC-MS analysis. To put it briefly, the study primarily concentrates on cultivated varieties, whereas research on wild resources remains minimal and represents a gap in the current research on BR.

With the development of molecular biology, it has become possible to explain the phylogeny and quality formation characteristics of authentic medicinal materials from the molecular level; especially, the development and application of genomics has become a powerful tool to decode the genetic causes of authentic medicinal materials. In recent years, the number of wild medicinal materials has gradually declined, especially YBC and YNC, which have become basically extinct in the market, and cultivated BR has become the main source of commercial BR. However, there are serious interspecific and intraspecific type-confounding phenomena, resulting in low yields and quality of BR. Therefore, it was necessary to domesticate wild understory Chinese medicinal materials manually and implement an ecological planting model [33,34,35]. In the process of a resource survey, the author found that there were large areas of YBC and YNC in Yiyuan County, Zibo City, Shandong Province. YBC was distributed in the shadow slope forest, and YNC was distributed in the sun slope. The growth environment of the two was quite different, and the growth rate was good. Based on this, we carried out extensive targeted metabolomics analysis based on UPLC-MS and GC-MS, systematically compared and identified metabolites in the two, and initially screened the different active components in YBC and YNC. The results show that there are significant differences in metabolite composition between the two, and these differences may affect their clinical application and efficacy. Understanding these differences is of great significance for guiding clinical drug use. The results provide a scientific basis for the biosynthesis pathway and related biological activities of BR components used in traditional Chinese medicine, which is of great significance for the utilization of wild BR medicinal resources and can promote the scientific utilization and sustainable development of traditional Chinese medicine resources [36]. This study found that the different metabolites of YBC and YNC were mainly flavonoids, phenolic acids, terpenes, etc., and provides a new way to study the wild resources of BR. The results of KEEG enrichment analysis showed that the different metabolic pathways were flavonoid and flavanol biosynthesis, linoleic acid metabolism, arachidonic acid metabolism synthesis, sesquiterpenoid and triterpenoid biosynthesis, and linolenic acid metabolism synthesis. This is also the main reason why flavonoids, phenolic acids, and terpenes are different in their metabolites.

To sum up, it was crucial to investigate the functional significance of metabolites in terms of pharmacological effects or clinical performance [37]. SS was considered to be the main active component of BR. In order to further improve the scientific and reliability of the study, we plan to further study the pharmacological mechanism of flavonoids and terpenoids by combining multi-omics techniques, such as transcriptomics, proteomics, and metabolomics, in subsequent studies. In future studies, we will consider included plant samples from different age stages to more fully assess the effects of age on metabolite distribution. At the same time, this study has certain limitations because the quality of medicinal materials was affected by many factors. In future work, it will be essential to increase the sample size and take into account factors such as expanding the origins and harvesting periods.

## 5. Conclusions

In this study, we conducted a broadly targeted metabolomics analysis to elucidate the differential metabolites between *Bupleurum chinense* DC. and *Bupleurum scorzoneraefolium* Willd., two closely related species with distinct pharmacological properties. In the meantime, this is the first attempt to report the metabolomics of the wild resource BR in Shandong province, and provides a reference for the study of wild resources. We used YBC and YNC as research materials. The metabolite differences between them caused the differences in their clinical efficacy. Our findings provide valuable insights into the metabolic profiles of these plants, which can aid in understanding their therapeutic differences and guide future research and development. In conclusion, this study provides a comprehensive metabolomics analysis of YBC and YNC, revealing their distinct metabolic profiles and potential therapeutic applications. The results not only contribute to the understanding of these important medicinal plants, but also pave a way for future research and development in the field of traditional medicine.

## Figures and Tables

**Table 1:** Statistics of YBC and YNC metabolites.

	Total	Common	Difference	YBC	YNC
Phenolic acids	324	319	133	2(p-Coumaroylcaffeoyltartaric acid, 2,4,6-Trihydroxybenzoic acid)	3(Acropyrone, Leonoside F, 3,5-Dicaffeoylquinic acid)
Dx rTerpenoids	270	256	117	6(Shanzhiside methyl eter,3-Hydroxy-11-oxoolean-12-en-30-oic acid (18α-Glycyrrhetinic acid), 27,28-Dicarboxyl ursolicaid, Clerodendrin I, Achyranthoside D, Armatoside)	8(Ceanothenicaid, Semiaquilegin A, 13-Methyl-27-norolean-14-en-3-ol (Taraxerol), Nootkatol, Icariside B_1_, Lupane-20(29)-en-3-on- 28-oic acid, lada-8(17), 12-diene-15,16-dial, [6-[acetyloxy-[1-(furan-3-yl)-5-hydroxy-8a-methyl-3,6-dioxo-7,8-dihydro-1H-isochromen-5-yl]methyl]-3-(2-methoxy-2-oxoethyl)-2,2,4-trimethyl-5-oxocyclohexyl]2-methylbutanoate)
Flavonoids	254	247	138	5(Quercetin-5-O-glucuronide, Dihydrocharcone-4′-O-glucoside, Genistein-8-C-glucoside-O-apiosyl, Luteolin-7-O-(6″-sinapoyl)glucoside, Quercetin-3-O-(2″-O-Xylosyl rutinoside)	2(Diosmetin-8-C(2″-Orhamnosyl) glucosde, Luteolin-3′-O-glucoside)
Amino acids and derivatives	192	192	38		
Lipids	191	191	41		
Alkaloids	176	173	42	3(Ethy11,2-dihydro-1-methyl-2-oxoquinoline-4-carboxylate, Caffeoylcholine-3-O-glucoside, Caffeoylcholine-4-O-glucoside)	
Lignans and Coumarins	173	172	81	1(5′-Methoxylariciresino1)	
Organic acids	120	118	28	1(Pyrrole-2-carboxylic acid)	1(Dihydrojasmonic acid)
Nucleotides and derivatives	72	72	10		
Quinones	16	16	8		
Tannins	3	3			
Others	268	267	82	1(Senkyunolide I)	
Total	2059	2026	718	19	14

**Table 2:** Metabolites in the top 20 of YBC and YNC roots with different multiples.

No.	Compounds	Class I	Class II	Formula	log_2_FC	Type
1	3,5-dihydroxy-4-{[(2s,3r,4s,5s,6r)-3,4,5-trihydroxy-6-[(sulfooxy)methyl]oxan-2-yl]oxy}benzoic acid	Phenolic acids	Phenolic acids	C_13_H_16_O_13_S	12.45	up
2	3,5-dihydroxy-4-[(2S,3R,4S,5S,6R)-3,4,5-trih-droxy-6-(sulfooxymethyl)oxan-2-yl]oxybenzoic acid	Phenolic acids	Phenolic acids	C_13_H_16_O_13_S	12.89	up
3	5-O-Feruloylquinic acid	Phenolic acids	Phenolic acids	C_17_H_20_O_9_	7.21	up
4	Kaempferol-3-O-rhamnoside (Afzelin)(Kaempferin)	Flavonoids	Flavonoids	C_21_H_20_O_10_	8.32	up
5	8-Methoxykaempferol-7-O-rhamnoside	Flavonoids	Flavonoids	C_22_H_22_O_11_	7.17	up
6	Kaempferol-7-O-rhamnoside	Flavonoids	Flavonoids	C_21_H_20_O_10_	8.48	up
7	Hispidulin-7-O-(6″-O-p-Coumaroyl)Glucoside	Flavonoids	Flavonoids	C_31_H_28_O_13_	7.02	up
8	Quercetin-3,7-Di-O-rhamnoside	Flavonoids	Flavonoids	C_27_H_30_O_15_	6.82	up
9	5,7-Dimethoxycoumarin (Limettin)(Citropten)	Lignans and Coumarins	Coumarins	C_11_H_10_O_4_	7.98	up
10	6,7-Dimethoxy-4-methylcoumarin	Lignans and Coumarins	Coumarins	C_12_H_12_O_4_	9.81	up
11	3-Methyl-6-methoxy-8-hydroxy-3,4-dihydroisocoumarin	Lignans and Coumarins	Coumarins	C_11_H_12_O_4_	8.21	up
12	Eugenin	Others	Others	C_11_H_10_O_4_	8.67	up
13	leptorumol	Others	Others	C_11_H_10_O_4_	8.23	up
14	30-O-Aangeloylhamaudol	Others	Chromone	C_20_H_22_O_6_	8.40	up
15	Vanillin acetate	Phenolic acids	Phenolic acids	C_10_H_10_O_4_	−7.62	down
16	6-Methylaloe emodin	Quinones	Anthraquinone	C_16_H_12_O_5_	−6.28	down
17	Hydroxystemofoline	Alkaloids	Pyrrole alkaloids	C_22_H_29_NO_6_	−7.52	down
18	Cinnamoyltyramin	Alkaloids	Phenolamine	C_17_H_17_NO_2_	−6.35	down
19	Ajugamacrin C*	Terpenoids	Diterpenoids	C_34_H_50_O_11_	−6.60	down
20	Ajugamacrin D*	Terpenoids	Diterpenoids	C_34_H_50_O_11_	−7.17	down

**Table 3:** Statistical information table of KEGG pathway enrichment in the group.

KEGG Pathway	Ko_ID	Number of Metabolites	*p*-Value
Flavone and flavanol biosynthesis	ko00944	13	0.0001
Linoleic acid metabolism	ko00591	9	0.0033
Arachidonic acid metabolism	ko00590	3	0.0388
Sesquiterpenoid and triterpenoid biosynthesis	ko00909	2	0.0518
alpha-Linolenic acid metabolism	ko00592	5	0.1133
Stilbene, diarylheptanoid and gingerol biosynthesis	ko00945	2	0.1321
Starch and sucrose metabolism	ko00500	3	0.1988
Monoterpenoid biosynthesis	ko00902	1	0.2285
Caffeine metabolism	ko00232	2	0.3217
Isoflavonoid biosynthesis	ko00943	2	0.3217
Biosynthesis of secondary metabolites	ko01110	38	0.3249
Glutathione metabolism	ko00480	4	0.3429
Carotenoid biosynthesis	ko00906	1	0.4052
Diterpenoid biosynthesis	ko00904	1	0.4052
Biosynthesis of various plant secondary metabolites	ko00999	6	0.4341
Flavonoid biosynthesis	ko00941	4	0.5166
Phenylpropanoid biosynthesis	ko00940	4	0.5166
Biotin metabolism	ko00780	1	0.5418
Indole alkaloid biosynthesis	ko00901	1	0.5418
Plant hormone signal transduction	ko04075	1	0.5418

## Data Availability

The original contributions presented in the study are included in the article. Further inquiries can be directed to the corresponding authors.
